# Emotional and Behavioural Problems among Preschool Children in Northeast Peninsular Malaysia: Parent Report Version

**DOI:** 10.3390/healthcare11131828

**Published:** 2023-06-22

**Authors:** Mohamad Hazni Abd Rahim, Mohd Ismail Ibrahim, Azriani Ab Rahman, Najib Majdi Yaacob, Nor Syuhada Farhanis Hashim

**Affiliations:** 1 Department of Community Medicine, School of Medical Sciences, Universiti Sains Malaysia, Kubang Kerian 16150, Kota Bharu, Kelantan, Malaysia; drmohamadhazni@student.usm.my (M.H.A.R.); azriani@usm.my (A.A.R.); 2 Unit of Biostatistics and Research Methodology, School of Medical Sciences, Universiti Sains Malaysia, Kubang Kerian 16150, Kota Bharu, Kelantan, Malaysia; najibmy@usm.my; 3 Unit of Psychology Counselling, Bachok District Health Office, Kelantan State Health Department, Ministry of Health Malaysia, Bachok 16300, Kelantan, Malaysia; syuhadafarhanis@gmail.com

**Keywords:** preschool children, behavioural problems, emotional problem, strengths and difficulties questionnaire, factor associated

## Abstract

The rising prevalence of mental health disorders among children is a serious concern. Young children who exhibit early warning signs of mental health issues are more likely to develop symptoms in the same or overlapping regions years later. The research aimed to identify emotional and behavioural problems and associated factors in Malaysian preschools. A sample of young children aged 4–6 years from public and private preschools was chosen using a multistage random sampling method. Data were collected from 18 preschools via a parent survey using the Strengths and Difficulties Questionnaire (SDQ). The sample involved 557 children in the SDQ assessment (92%). The overall estimated prevalence of emotional and behavioural problems was 8.4%. Peer problems were the most prevalent attribute, with a percentage of 19.7%. Conduct problems were found in 5.2%, hyperactivity problems in 5.6%, prosocial behaviours in 13.5%, and emotional problems in 6.8%. Girls showed a significant increase in behavioural and emotional problems compared to boys. Having one parent working, having more than two siblings, and having a single-parent family were associated with emotional and behavioural problems. The prevalence of emotional and behavioural problems in Malaysian children was relatively low compared to data from previous studies and other Asian countries but consistent with European studies. Measuring mental health disparities in young children helps stakeholders launch local early intervention programmes.

## 1. Introduction

The preschool period is the essential stage of life when major development creates the foundation and determines the course of later life. Children’s behaviours vary, and there is no clear separation between problematic behaviour and a serious emotional problem. A child can develop a specific “problem” or “disorder” if his or her behaviours are frequent and severe. The most common psychosocial health problems in preschool children are emotional and behavioural problems (EBD) [1]. Children with behavioural and emotional problems in preschool are more likely to experience mental health challenges throughout childhood and adolescence [2]. However, many preschool children are not diagnosed and do not receive the required support. These children have a high risk of developing serious psychiatric disorders, poor social skills, and educational difficulties in the future if they are not diagnosed and treated early on [3].

Mental health disorders (mental disorders and mental illnesses) are significant in children and adolescents, with a 2015 meta-analysis estimating a worldwide prevalence of 13.4% [4]. According to a 2011 study, the global prevalence of mental health problems among children ranges from 10% to 20% [5]. In the past decade, there has been an increase in the global prevalence of diseases regarded as serious public health concerns. In a review, Teekavanich and Chantaratin [6] discovered that the prevalence of emotional and behavioural problems among preschoolers ranged from 7% to 25% for epidemiological studies of psychopathology using checklist measures. Over the past few decades, the progress in the prevalence of emotional and behavioural problems in different countries and cultures has reflected demands on youth mental health worldwide [7].

In most European nations, the prevalence of preschool children ranged from 6.3% to 9.8% [8]. The prevalence of mental health illnesses among preschool children varies among Asian countries. The prevalence of mental health problems was 11.1% among 3–4-year-olds in Bangladesh [9], 11.9% among preschool children 4–6 years in Bangkok, Thailand [6], and 13.6% among preschool children 3–6 years in China [10]. According to the National Health and Morbidity Survey (NHMS) conducted in Malaysia, there was an increasing tendency for mental health disorders among children aged 5–15 years from surveys conducted in 1996 (13.0%), 2006 (19.4%), 2011 (20.0%), and 2015 (12.1%) [11]. Previous Strength and Difficulty Questionnaire (SDQ)-based studies in Malaysia revealed a 16.4% prevalence of mental health problems in the urban setting of Negeri Sembilan [12] and a 16.2% prevalence in Selangor among 5–13-year-olds [13]. Previous research also found that peer difficulties were the most prevalent SDQ subscales in national surveys [11] (31.0%), Negeri Sembilan [12] (44.0%), and Selangor [13] (23.7%).

Understanding the factors associated with emotional and behavioural problems is vital for developing targeted intervention strategies. Previous local studies demonstrated that boys’ and fathers’ education was significantly associated with emotional and behavioural problems [14]. Parents with a higher level of education frequently have a better understanding of child development and access to resources that help them improve their parenting abilities. Compared to girls, most studies found boys to be more at risk of developing emotional and behavioural problems [14,15,16]. Many studies have highlighted socioeconomic status or household income as a significant factor associated with emotional and behavioural problems in preschool [17,18,19]. Lower household income frequently correlates with higher levels of socioeconomic stress and hardship, which can lead to a less caring and nurturing environment for children. Other preschools’ behavioural and emotional risk factors include parental divorce, family conflicts, child health issues, parental mental health, first childbirth, having siblings, parent ages, rural or urban living, and working partners in the family [6,11,18,20,21,22,23].

There was limited local research on preschool emotional and behavioural problems, with only two studies to date. Previous research was conducted along the western coast of Peninsular Malaysia in its most urbanised and developed state [12,14]. Kelantan, located on the eastern coast of Peninsular Malaysia and predominately rural in nature, has a unique sociocultural and socioeconomic context that may influence the prevalence and factors contributing to emotional and behavioural problems in preschool children. Access to economic, environmental, cultural, and service opportunities may vary between rural and urban families. There is a need for localised research to gain a deeper understanding of the unique challenges children face in Kelantan, as the prevalence rates of these problems and their associations may vary by region and population.

Numerous studies describe the prevalence of mental health disorders in school-aged children, adolescents, and adults, but fewer studies focus on preschoolers [24]. Thus, this study aimed to determine the prevalence and scores of emotional and behavioural problems in Malaysian preschoolers and identify factors associated with emotional and behavioural problems. The study’s results may have significance for academics and practitioners in early childhood development, psychology, and early education. Policymakers can develop targeted interventions to promote healthy development and well-being among preschool children in Kelantan by identifying the prevalence and factors associated with behavioural and emotional problems, ultimately contributing to the overall improvement of early childhood services and support systems.

## 2. Materials and Methods 

### 2.1. Study Design and Participants

A cross-sectional study was conducted among preschool children aged 4–6 years in Kelantan, Malaysia’s East Coast. Participants were recruited, and data were collected at selected public and private preschools in two districts of Kelantan State between November and December 2022. The sample size required for this study was 612, which was calculated using a single-proportion calculation with an expected sample size of 20% dropout, a margin of error of 5%, a confidence interval of 95%, a prevalence of behavioural problems among preschool children of 15% [25], and a design effect of 2.5.

This research employed a multistage random sampling technique. Initially, each of the nine districts was randomly selected after being stratified into urban and rural regions. In the second phase of sampling, stratified proportional random sampling was applied. The total number of public and private preschools in each district (*n* = 671) was used to select five public and four private preschools at random (2%). In the final stage, each of the 18 preschools was stratified into 4–6-year-old classrooms. The final stage was selected by simple random sampling. All eligible parents of children aged four, five, and six were recruited as research participants, with an estimated average of 34 per preschool. Parents were invited to act as proxies for their children in this study. Only parents of Malaysian children who can understand and write in Bahasa Malaysia were included. Children who had already been diagnosed with any developmental disorders or were on extended medical leave were excluded.

Principals and teachers at the participating preschools received a detailed summary of the study. With the principal’s permission, the teachers were asked to distribute via envelopes to the parents of 4–6-year-olds a letter of invitation describing participant information, a form of informed and voluntary consent to participate in the study, and research questionnaires. If the parents agreed to participate, they gave written consent, answered the Malay version of the SDQ questionnaire, and provided sociodemographic information about themselves and their child. The questionnaires and consent forms were returned to the teachers in envelopes, which the researcher subsequently collected.

### 2.2. Measurements

The Strengths and Difficulties Questionnaire (SDQ) was used to assess the emotions and behaviour of preschool children [26]. SDQ is one of the most widely used and translated screening measures of child mental health in the community, having been translated into over 60 languages [27]. The parents or guardians of the respective children in this study completed the SDQ. The Malay version of the SDQ is reliable and valid. Cronbach’s alphas for the Malay version of the parent-reported SDQ were 0.76, and confirmatory factor analysis (CFA) revealed that the original five-factor model fit well [28].

Parents were asked to rate 25 items, which comprised 5 subscales: hyperactivity, emotional symptoms, conduct problems, prosocial behaviours, and peer problems. Each item received 3 points: 0 for not true, 1 for somewhat true, and 2 for definitely true. A higher score indicated more problems across the subscales of the SDQ, except for the prosocial behaviour subscale, where a lower score indicated more problems [29]. The five scales are scored from 0 to 10 and classified as “normal”, “borderline”, or “abnormal”. Data scoring and categorization were carried out following the guidelines provided by the instruments [30].

The total difficulty score was obtained using the scores from each subscale except “prosocial behaviours”, and the result score ranged from 0 to 40. A higher score indicates an increased need for help and support. The SDQ total difficulty scores are banded as ‘normal’ (0–13), ‘borderline’ (14–16), and ‘abnormal’ (17–40). Children with total difficulty scores of “borderline” or “abnormal” were grouped together and considered a “risk group” for emotional and behavioural health problems. As a result, if the total difficulty score was 14 or higher, children were considered to have psychological disorders [29,31]. Five SDQ subscales were also grouped and assigned to the ‘risk group’ for the clinical cut-off, including everyone who scored borderline or higher. Cut-off scores are recommended for Malaysian children to assess children at high risk of emotional and behavioural problems (conduct problems > 3, hyperactivity problems > 5, peer problems > 3, prosocial behaviours > 6, emotional symptoms > 4, and total difficulties > 14) [32].

### 2.3. Statistical Analysis

The data were cleaned and coded using Microsoft Excel and analysed using SPSS version 26.0. In the study, we first showed the distribution of general characteristics and then examined the prevalence of behavioural problems. Categorical variables were expressed as a number (%), and continuous variables were expressed as mean ± standard deviation. Next, in the descriptive analysis, we reported the prevalence of emotional and behavioural problems measured by the Strengths and Difficulties Questionnaire. The means of the total difficulties and subscale SDQ scores and the proportions of children with normal, borderline, and abnormal scores were reported. The Risk group for emotional and behavioural problems (borderline and abnormal) served as the dependent variable to determine the associated factor. Simple and Multiple logistic regressions were done to examine the association between the independent variables and emotional and behavioural problems. As the dependent variable was dichotomous, we used a logistic regression model to produce crude odds ratios (ORs) to measure association. The adjusted ORs, with their respective 95% confidence intervals (CIs), were then calculated. A *p*-value of less than 0.05 (two-tailed) was selected as statistically significant.

## 3. Results

A total of 612 questionnaires were distributed, of which 557 were completed for a response rate of 91%, and the majority of informants were mothers (66.3%). The average age of the children was 70.19 months (SD = 7.94); 52.1% were boys, and 47.9% were girls. The percentages for the 4-year, 5-year, and 6-year groups were 5.7%, 37.5%, and 56.7%, respectively. Only the morning preschool session was attended by 91% of the children. Most preschoolers (70.9%) had at least two siblings, and 40% were born in the middle of the family. Regarding the characteristics of the parents, the majority of participants were female (66.6%), married (96.2%), and had completed secondary school (65.9%). Most of the children (40.2%) belonged to households with incomes in the bottom group, 40%, followed by those with poor incomes (35.7%). Further, 54.8% of families only had one working partner, and 29.1% were housewives, followed by the self-employed (27.1%) and government employees (24.2%). The predominant family types were nuclear families (71.8%) and extended families (26.4%). The sociodemographics of the participants are summarised in Table 1.

The prevalence and mean scores of emotional and behavioural problems within the normal and clinical range (risk) are listed in Table 2. The prevalence of emotional and behavioural problems was 8·4% of the population. The majority of subscales of the SDQ were reported in the current study, with 110 (19.7%) reporting peer problems, 75 (13.5%) reporting prosocial behaviours, 38 (6.8%) reporting emotional problems, 31 (5.6%) reporting hyperactivity problems, and 2.9 (5.2%) reporting conduct problems.

Univariable analyses (Table 3) revealed that nine variables, including parent age, female children compared to male children, children with more than two siblings compared to children with one sibling, firstborn child compared to middle birth, primary and secondary parent education level compared to tertiary education, widowed compared to married, single parent family compared to the nuclear family, and poor household income compared to the top 20% income level, were associated with emotional and behavioural problems (*p*-value < 0.25). No significant effects were found on total difficulty scores for the age of children, class year, locality of preschool, or parent marital status. When the multiple logistic regression was run (Table 3), four of the nine variables significantly contributed to the model. The analyses revealed that, compared to boys, girls significantly increased their total difficulty scores by 1.89 times (aOR: 1.89, 95% CI: 1.01, 3.56). Compared to having both parents working, one parent working (aOR: 2.06, 95% CI: 1.03, 4.09) was 2.06 times more likely to have emotional and behavioural problems. Furthermore, having more than two siblings (aOR: 0.38, 95% CI: 0.19, 0.73) was a protective factor compared to having only one sibling. Besides that, those living in a single-parent family were 6.04 times more likely to have emotional and behavioural problems than those living in a nuclear family (aOR: 6.04, 95% CI: 1.58, 23.15).

Table 4 presents the chi-square test analysis of selected variables with partners working on risk factors of emotional and behaviours problems. The findings show that education level and household income are significantly (*p*-value = 0.05) associated with the partner working on risk factors of emotional and behavioural problems among respondents.

## 4. Discussion

Mental health studies conducted in large population-based studies previously revealed that mental health problems were higher among younger children, making it imperative to investigate the prevalence and risk factors for emotional and behavioural disorders among preschoolers. The prevalence of emotional and behavioural problems in this study was 8.4%, which is lower than the previous study in urban Negeri Sembilan preschool [12] (16.4%), Selangor 5–13-year-olds [32] (11.2%), the National large population study of Malaysian children 5–15-year-olds [32] (11.1%), and other preschool age Asian countries in Bangladesh [9] (11.9%), Thailand [6] (11.9%), and China [10] (13.6%). However, study results are consistent with the prevalence of 6.3–9.8% in most European countries [8]. The substantial difference between the results of this study and those of other studies reported in this article may be attributable to several factors, including sample characteristics, measurement techniques, and cultural differences. For example, in a large population study of Malaysian children [11], cut-off measures from the UK were used, with total difficulties SDQ > 14 [29] compared to our cut-offs, which were >15. Previous research by Farhana [13] discovered that the UK cut-off scores proposed by Goodman were overinclusive for the Malaysian population in peer problems, conduct problems, emotional symptoms, and total difficulties. According to Rescorla and Achenbach [33] any comparison of the prevalence of psychiatric disorders is affected by various factors, most notably the use of different methodologies, measures, and ascertainment techniques.

The disparity between the findings of this study and those reported here could be attributed to parental and cultural differences. Our research in Kelantan, a rural area in Malaysia, differed from previous studies in that it was rich in religious and traditional cultural influences. Previous studies have found that Malaysian parents have higher standards and expectations for their children in urban areas, which may explain the high level of behavioural problems reported by parents. Our findings, in contrast with Farhana [13], show that overrated scores of behavioural problems can be attributed to Malaysian parents being more strict with their children. In addition to the fact that technology and media use has been influencing parent-child interactions and parental engagement contributes to fewer parents underreporting current symptoms, parents may find it difficult to assess the emotional behaviour of preschool-aged children. Additional factors, according to Van Roy and Groholt [34], may have contributed, such as a decrease in parents underreporting current symptoms and equal access to social, technological, and educational facilities.

In terms of SDQ subscales, our findings show that peer problems were reported in (19.7%), followed by prosocial behaviours (13.5%), emotional problems (6.8%), hyperactivity problems (5.6%), and conduct issues (5.2%). In previous studies, Malaysia’s national survey [11] (31.0%), Negeri Sembilan [12] (44.0%), Selangor [13] (23.7%) and China [10] (25.5%) reported that the highest prevalence of subscale was peer problems. Previous research findings and our findings have indicated that Malaysian children have a peer problem that needs to be addressed. Peer problems occur when preschool children have difficulty cooperating with others and making friends. Enduring peer relationship problems as a child has been found to predict internalised issues such as isolation, depression, anxiety, physical health, and school difficulties [35].

Cultural perspectives may significantly impact children’s experiences with peer relationships. For instance, the dominance of shyness-inhibition cultural values in Malaysian culture since childhood may influence peer attitudes towards shy-inhibited children during interactions. When shy-inhibited children initiated interplay, typically applying a passive and low-power approach, their peers were likely to reject and ignore them [36]. Asian children were more accepting of shy and wary behaviours than children in Western countries such as Australia, Germany, the Netherlands, and the United States [37]. Young children are believed to transfer behavioural and relationship patterns learned from their families, such as peer relationships, to their peers. Positive parenting (i.e., authoritative parenting, interaction, engagement, and support) was associated with a small to moderate decline in peer relationship problems (bullying and victimisation), whereas negative parenting (i.e., abuse and abandonment, maladaptive parenting, and overprotection) was associated with an increase in these problems [38].

However, we cannot deny that children’s behavioural characteristics have contributed to these peer issues. Children that struggle with peer relationships are typically more violent, hyperactive, and rebellious, but they are also more socially withdrawn and less friendly. Aggressive behaviours are the most commonly reported behavioural correlates and primary causes of peer rejection in school settings [35]. Our data also revealed that prosocial behaviour was the second most prevalent and linked to peer difficulties. Children who demonstrate prosocial behaviour and social competence at a young age are more likely to be accepted by their peers. Aggressive and antisocial preschoolers generate fewer ideas than their typically developing friends [39].

The present study revealed a significant association between emotional and behavioural problems among boys and girls, with girls exhibiting a higher risk than boys. This finding is consistent with the other studies [40]; however, it was contradicted by a previous study that found boys at greater risk than girls [14,15,16]. This result may be explained by the fact that children feel anxious and fearful upon entering a new preschool classroom following a lengthy stay at home and movement restrictions during the COVID-19 pandemic. Manifested more frequently in girls than in boys, these symptoms may be indicative of an internalising issue (such as anxiety). Young girls are more susceptible to anxiety than boys [41]. Consequently, public health and preschools should be aware of the possibility that gender differences in children’s needs reflect their different characteristics.

A significant association was found between having one parent working and a higher occurrence of emotional and behavioural problems compared to having both parents working. The findings contradict our initial assumption that both working parents who are unable to spend enough time with their children are at a higher risk of emotional and behavioural problems. According to the findings, one parent working with a low education level and a low household income was associated with an increased risk of emotional and behavioural issues. Similarly, the higher the parent’s education level and household income, the lower the risk of emotional and behavioural issues. There could be several explanations for this pattern. Families with two working parents may experience less conflict and fewer depressive symptoms because better earnings prospects lower the possibility of financial difficulties. In addition, good facilities and child-friendly policies in Malaysian preschools reduce work-family conflict. Single-income working families tend to have more depressive symptoms than dual-income working families due to having a better income and policies that support child benefits [23]. Higher-educated parents are more aware of good parenting and have compensated with the quality of time spent with their children. Children with full-time working mothers spend approximately six fewer hours per week in unstructured play activities than children with stay-at-home mothers, which would positively influence children’s growth [42].

A recent study also discovered that children with more than two siblings have fewer emotional and behavioural problems than children with only one sibling. In line with Hughes and Ronchi [43], children with one or more older siblings had more emotional and behavioural problems than children without siblings. One possible explanation is that healthy sibling relationships can be a tremendous source of support, promote empathy, and ensure that siblings have a strong support system, which helps them avoid developing mental health challenges themselves. Sibling relationships can improve self-regulation and emotional understanding by providing positive support and skill development [44]. However, research on children with one or more siblings is limited and inconsistent.

In addition, these findings contribute to our knowledge of the evidence showing children living in a single-parent home are considerably more likely to have emotional and behavioural difficulties than children living in a nuclear family. This result might be attributed to the fact that the majority of single parents in our sample were divorced and from low- or lower-middle-income families. Single parents with low socioeconomic standing are frequently confronted with financial difficulties, limited resources, and increasing economic strain. Financial hardship and economic stressors can lead to parental distress, impacting the parent-child connection and raising. Previous research has shown that children from single-parent families have higher behavioural problems than children from nuclear families, which are associated with divorce, parent conflicts, financial difficulties, parental mental health problems, and poor parenting quality [45].

### 4.1. Limitation

One limitation of the study is that mental health assessments are based on participant self-report, which may lead to a lack of understanding and interpretation of the items and response bias. Parents are the most familiar with their children, but their own subjective needs may bias them. The evaluations were conducted by preschool parents, which is insufficient because a detailed assessment of the factor structure should also include teacher assessments or clinical observation by psychiatric or developmental experts.

The second limitation of our study is the method we used to select our sample. Due to time, budget, and logistical constraints, this study will only be conducted in two districts and will involve eighteen Kelantan preschools, not other care centres. On the other hand, using multistage random sampling throughout the selection method may help reduce sample bias and increase the generalizability of the results. As a result, the findings can be applied to children in Kelantan preschools. Furthermore, causality cannot be concluded because this study will employ a cross-sectional design.

### 4.2. Recommendation

Despite the limitations mentioned above, this research undoubtedly makes a scientific contribution to several different research areas from a methodological standpoint. Determining the profiles of SDQ emotional and behavioural problems and their associated factors in preschool children may enable teachers, parents, and other responsible parties to quickly identify and assist students experiencing socio-emotional difficulties. As a result, the prevention and treatment of mental illnesses in children should be top priorities. Screening for mental health problems in at-risk children is a standard of care. More research with larger sample sizes from multiple regions in Malaysia using multiple informants (both the informant’s parent and the teacher) is required.

## 5. Conclusions

This study reveals that the prevalence of emotional and behavioural problems measured by the Strengths and Difficulties questionnaire was 8.4%, with peer problems being the most common. Female gender, one parent working, having more than two siblings, and a single-parent family were associated with a higher risk of emotional and behavioural problems among preschool children aged 4–6 years. Even though the prevalence was lower than that in previous studies, peer problems remain a priority for the stakeholders by providing information about this issue and encouraging related organisations to create useful intervention and prevention initiatives. Enduring peer relationship problems as a child has been found to predict internalised issues such as isolation, depression, anxiety, physical health, and school difficulties.

## Figures and Tables

**Table 1:** Study participant sociodemographics in preschool children (*n* = 557).

Variables	Variable Characteristics	*n* (%)
Age of children (months) *		70.19 (7.94)
**Child’s class year**	4 years	32 (5.7)
	5 years	209 (37.5)
	6 years	316 (56.7)
**Locality**	Rural	272 (48.8)
	Urban	285 (51.2)
**Type of preschool**	Public	301 (54)
	Private	256 (46)
**Preschool session**	Morning and transit or other care centres	50 (9)
	Morning session	507 (91)
**Child’s gender**	Girls	267 (47.9)
	Boys	290 (52.1)
**Month of pregnancy**	Term delivery	548 (98.4)
	Premature	9 (1.6)
**Underlying medical condition**	No	531 (95.3)
	Yes	26 (1.6)
**Number of siblings**	Only child	34 (6.1)
	With 1 sibling	128 (23)
	With ≥2 siblings	395 (70.9)
**Birth order**	Firstborn	148 (26.6)
	Middle	223 (40)
	Last born	186 (33.4)
**Informant relationships**	Mother	369 (66.3)
	Father	184 (33)
	Guardian relation	4 (0.7)
Age of parent (years) *		36.84 (5.96)
**Parent gender**	Female	371 (66.6)
	Male	186 (33.4)
**Education level**	Primary	21 (3.8)
	Secondary	367 (65.9)
	Tertiary	169 (30.3)
**Marital status**	Married	536 (96.2)
	Divorced	12 (2.2)
	Widowed	9 (1.6)
**Working**	Housewife/Not working	162 (29.1)
	Government	134 (24.1)
	Private	90 (16.2)
	Self-employed	151 (27.1)
	Farmer/breeder	20 (3.6)
**Partner working**	Both parents working	252 (45.2)
	One parent working	305 (54.8)
**Household income**	Poor < MYR 1000	199 (35.7)
	Bottom 40%	224 (40.2)
	Middle 40%	96 (17.2)
	Top 20%	38 (6.8)
**Family type**	Nuclear family	400 (71.8)
	Extended family	146 (26.4)
	Single-parent family	11 (1.8)

* Mean (±SD).

**Table 2:** Prevalence and mean scores of behavioural and emotional problems among preschool children (*n* = 557).

SDQ	Scores (Mean ± SD)	Normal *n* (%)	Risk/Clinical Cut-Off *n* (%)
Total difficulties SDQ ^1^	8.29 ± 4.12	510 (91.6)	47 (8.4)
Subscales of the SDQ ^2^			
Emotional symptoms	1.77 ± 1.60	519 (93.2)	38 (6.8)
Conduct Problems	1.41 ± 1.21	528 (94.8)	29 (5.2)
Hyperactivity Problems	2.72 ± 1.70	526 (94.4)	31 (5.6)
Peer Problems	2.39 ± 1.38	447 (80.3)	110 (19.7)
Prosocial behaviour	7.72 ± 1.75	482 (86.5)	75 (13.5)

^1^ Total Difficulty scores range from 0 to 40 (except prosocial behaviour). ^2^ Scores in each subscale range from 0 to 10.

**Table 3:** Multiple logistic regression analysis of factors sociodemographic and prediction of total difficulty scores.

	Univariable			Multivariable		
Variable	Crude OR ^1^	95% CI ^1^	*p*-Value	Adjusted OR ^1^	95% CI ^1^	*p*-Value
**Age of parent**	0.95	0.90, 0.99	0.046			
**Child’s gender**						
Boys	1			1		
Girls	1.84	0.99, 3.40	0.051	1.89	1.01, 3.56	0.048
**Number of siblings**						
Only child	0.73	0.26, 2.59	0.728	0.61	0.18, 2.00	0.411
With 1 sibling	1			1		
With ≥2 siblings	0.41	0.22, 0.79	0.007	0.38	0.19, 0.73	0.004
**Birth order**						
Firstborn	2.04	1.00, 4.16	0.049			
Middle	1					
Last born	1.04	0.48, 2.25	0.917			
**Education level**						
Primary	2.96	0.74, 11.95	0.127			
Secondary	1.87	0.88, 3.99	0.104			
Tertiary	1					
**Partner working**						
Both parent working	1			1		
One parent working	2.31	1.19, 4.47	0.013	2.06	1.03, 4.09	0.039
**Marital status**						
Married	1					
Divorced	2.29	0.49, 10.80	0.294			
Widowed	3.28	0.66, 16.26	0.147			
**Family type**						
Nuclear family	1			1		
Extended family	1.36	0.70, 2.65	0.335	1.13	0.56, 2.26	0.734
Single-parent family	7.31	2.02, 26.43	0.002	6.04	1.58, 23.15	0.009
**Household income**						
Poor < MYR 1000	2.59	0.59, 11.41	0.210			
Below 40%	1.48	0.33, 6.67	0.611			
Middle 40%	0.58	0.09, 3.62	0.560			
Top 20%	1					

^1^ OR = Odds Ratio, CI = Confidence Interval. Constant = −2.64; Backward LR method was applied; No multicollinearity and no interaction; Hosmer Lameshow Test of fit, *p*-value = 0.054 (>0.05); Classification table 91.7% correctly classified; Area under Receiver Operating Characteristic (ROC) curve was 66.4%, *p*-value ≤ 0.05.

**Table 4:** Chi-square test of association between partner working categories and selected study variables.

Variable	Partner Working			
	Both Parent Working *n* (%)	One Parent Working *n* (%)	χ^2^	*p*-Value
**Education level**			64.13	<0.001
Primary	4 (1.6)	17 (5.6)		
Secondary	129 (51.2)	367 (78)		
Tertiary	119 (47.2)	50 (16.4)		
**Household income**			136.06	<0.001
Poor < MYR 1000	39 (15.5)	160 (52.5)		
Below 40%	100 (39.7)	124 (40.7)		
Middle 40%	79 (31.3)	17 (5.6)		
Top 20%	34 (13.5)	4 (1.3)		

## Data Availability

The data are not publicly available due to privacy and confidentiality. However, restrictions apply to the availability of hospital data and are available from the authors with the permission of the organisation.
